# IMplementation of Realist Findings to Create an Innovative Pathway for Weight Gain Associated With Anti-Psychotic Use in Patients Living With Severe MENTal Illness (SMI) – IMPLeMENT

**DOI:** 10.1192/bjo.2025.10121

**Published:** 2025-06-20

**Authors:** Jasleen Deol, Hafsah Habib, Ian Maidment, Ita Fitzgerald, Jo Howe

**Affiliations:** ^1^Black Country Healthcare NHS Foundation Trust, Dudley, United Kingdom; ^2^School of Pharmacy, Aston University, Birmingham, United Kingdom; ^3^St Patrick’s University Hospital, Dublin, Ireland; ^4^School of Pharmacy, University College, Cork, Ireland

## Abstract

**Aims:** Antipsychotic medications are essential in treating patients with severe mental illness (SMI), but they are associated with rapid weight gain, significantly increasing the risk of cardiovascular disease and diabetes. These physical health complications contribute to reduced life-expectancy and development of preventable physical health conditions. The NIHR-RESOLVE study (REF:HSDR131871) explored non-pharmacological interventions for antipsychotic induced weight gain, and highlighted the urgent need for a structured, preventative pathway to support patients at risk.

Fragmentation between physical and mental health services, can leave patients with SMI without adequate physical health support. Despite national guidelines recommending annual physical health checks, interventions often come too late, typically after significant weight gain has already occurred. Additionally, stigma surrounding weight gain exacerbates mental health difficulties.

**Methods:** IMPLeMENT, an impact study funded by Aston University, facilitated three online workshops, and one in-person event to co-produce a person-centred preventative pathway to manage antipsychotic-induced weight gain. In collaboration with The McPin Foundation, sessions were attended by psychiatrists, dieticians, occupational therapists, policymakers, managers, commissioners, pharmacists and service users. Findings from RESOLVE served as a foundation for discussions, and healthcare professionals from the UK and international mental health services (including learning disabilities, forensic psychiatry, and early intervention in psychosis) shared experiences and discussed how they may implement change in local settings.

**Results:** Thematic analysis of workshop transcripts and notes revealed key challenges and opportunities in developing an effective preventative pathway. Stakeholders highlighted several areas for improvement:

Holistic approaches – current interventions are often fragmented, lacking integration across services.

Shared Responsibility – The need for collaborative care among different HCPs and services was emphasised.

Improved access to information – service users and professionals expressed the importance of psycho-education and clear, accessible resources to facilitate proactive management.

As a direct result of this work, multiple healthcare organisations are implementing and evaluating preventative weight management pathways tailored to their local needs and available resources. These quality improvement (QI) projects mark a significant step toward embedding sustainable change in routine care.

**Conclusion:** This work has emphasised the need for a novel preventative pathway to mitigate the risks of antipsychotic weight gain. Future research will work alongside these organisations to examine real-world implementation, identifying facilitators and barriers to integrating preventative pathways into everyday clinical practice. By doing so, this initiative aims to bridge the gap between physical and mental healthcare, improving long-term health outcomes for individuals living with SMI.

